# Coexisting genetic kidney disease explains many cases of ‘familial’ IgA nephropathy where the proband has biopsy-confirmed mesangial IgA deposits

**DOI:** 10.3389/fmed.2025.1647695

**Published:** 2026-03-02

**Authors:** YuXin Li, Jing Zhang, Zhuo Jun Yin, Ziming Zhou, Bocheng Huang, Khalid Mahmood, Deb Colville, David Barit, Russell Auwardt, Robert Fassett, Kathy Paizis, Francesco Ierino, Timothy Pianta, David Langsford, Mary Huang, Judy Savige

**Affiliations:** 1 Department of Medicine, Melbourne Health and Northern Health, The University of Melbourne, Parkville, VIC, Australia; 2 Melbourne Bioinformatics, The University of Melbourne, Parkville, VIC, Australia; 3 Department of Surgery (Ophthalmology), Royal Victorian Eye and Ear Hospital, East Melbourne, VIC, Australia; 4 Renal Unit, Northern Health, Epping, VIC, Australia; 5 Renal Unit, Albury Hospital, Albury-Wodonga, VIC, Australia; 6 Tasmanian Health Service, North West, Burnie, TAS, Australia; 7 Department of Nephrology, Austin Health, Melbourne, VIC, Australia

**Keywords:** Alport syndrome, autosomal dominant, familial hematuria, familial kidney disease, genetic kidney disease, IgA nephropathy, severe IgA nephropathy

## Abstract

**Background:**

One in seven people with IgA nephropathy has another apparently-affected family member. This study examined how often biopsy-proven familial and sporadic IgA nephropathy were associated with genetic kidney disease.

**Methods:**

Eleven unrelated people with biopsy-proven IgA nephropathy and another family member with kidney tests compatible with IgA nephropathy were recruited. All available family members were assessed for genetic kidney disease, using Whole Exome Sequencing (WES). Their results were compared with those of 39 people with sporadic IgA nephropathy. All sequencing results were filtered for pathogenic variants in genes associated with genetic kidney disease (Genomics England panels, *n* = 384). Variants were assessed for pathogenicity using ClinVar and the ACMG/AMP criteria in Alamut.

**Results:**

Nine of the 11 probands (82%) with familial nephropathy and 30 of those with sporadic disease (77%) had kidney failure. At least five (45%) and possibly nine (82%) of the 11 families studied had disease-associated heterozygous variants consistent with a coexistent genetic kidney disease (autosomal dominant (AD) and X-linked (XL) Alport syndrome, ADTKD-*HNF1B*, and possibly Dent disease, Focal and Segmental Glomerulosclerosis (FSGS), and CHARGE syndrome). Inheritance for all these diseases was AD or X-linked. Sometimes the proband with IgA nephropathy did not have the genetic variant found in other apparently-affected family members. Sometimes two genetic variants corresponding to two different diseases were present in the same family. Two of the 39 people with sporadic IgA nephropathy (5%) also had disease-causing variants consistent with genetic kidney disease (AD Alport syndrome, cystinuria).

**Conclusion:**

Many familial cases of IgA nephropathy result from mesangial IgA deposition in the setting of coexisting genetic kidney disease. Sometimes, genetic kidney disease is not detected in the proband but is present in another family member. Individuals from families with IgA nephropathy should be offered genetic testing.

## Introduction

IgA nephropathy is possibly the commonest form of glomerulonephritis worldwide (1, 2). It is characterized by macroscopic hematuria associated with mucosal infections, and diffuse mesangial deposits of IgA, IgG, and C3. One in four of those affected develops kidney failure after 20 years (3), and IgA nephropathy accounts for 6% of patients requiring renal replacement therapy (4).

Our current understanding of the pathogenesis of IgA nephropathy includes ‘multiple hits.’ (5) Mucosal infections, including EBV (6) result in galactose-deficient IgA1 (7), which is targeted by anti-glycan IgG antibodies (8), forming immune complexes in the glomerular mesangium and activating complement by the alternative and lectin pathways (9). The diverse properties of these immune complexes (composition, molecular mass, etc.) may explain the presence or absence of symptomatology.

The evidence for genetic involvement in the pathogenesis of IgA nephropathy is complicated. Fifteen percent of people with biopsy-proven IgA nephropathy have another apparently-affected family member (10), often with disease in each generation. These familial forms of IgA nephropathy are typically more severe than sporadic disease, with earlier onset of kidney failure (11, 12). However, the familial association and increased severity are unexplained.

In IgA nephropathy, the abnormal mesangial IgA1 deposits are determined genetically by variants in the *C1GALT1* gene (13). These variants likely contribute to the increased disease susceptibility in people of East Asian and Southern European ancestries (14). However, the effect of the *C1GALT1* variants is unclear since only some family members with this change develop IgA nephropathy. Furthermore, while galactose-deficient mesangial IgA1 deposits are noted post-mortem in up to 20% of the population, many fewer people have an active glomerulonephritis (15–17).

Nevertheless, genetic variants that predispose to IgA nephropathy have been identified in potentially relevant pathways in various Genome Wide Association Studies (GWAS). These pathways affect mucosal innate immunity against pathogens (*DEFA* cluster, *PADI4, IRF4, UBR5, CARD9, PSMB8/PSMB9, FCRL3*); IgA1 production (*TNFSF13, LIF, OSM*) and glycosylation (*ST6GAL1*); antigen presentation (HLA cluster, *PSMB8/PSMB9*, *TAP1/TAP2*); lymphocyte maturation (DUSP22, VAV3); and complement activation (*CFH, CFHR3-CFHR1, ITGAM, ITGAX*) (18–21). HLA and additional genes have been implicated in IgA nephropathy and related diseases such as IgA vasculitis (formerly Henoch-Schonlein purpura) (22), Inflammatory Bowel Disease (23) and Ankylosing Spondylitis (24). In general, common variants in the GWAS-identified genes each have a small additive effect on disease risk, but rare deleterious variants in the same genes may have more pronounced consequences.

Finally, monogenic causes have been reported in some cases of familial IgA nephropathy. Affected genes include *SPRY2*, which encodes an inhibitor of B cell proliferation (25), as well as complement pathway genes (26, 27), and occasionally other apparently coincidental genetic kidney diseases (28–31). Up to 20% people with familial IgA nephropathy have been reported to have autosomal dominant (AD) or XL Alport syndrome, two of the most common genetic diseases affecting the kidney (32). Sometimes immunodeficiency genes are also associated with the development of autoimmune glomerulonephritis (33, 34).

This study examined how often familial causes were associated with an underlying genetic kidney disease, and differed from previous studies in that multiple family members were examined. These results were compared with those from people with sporadic IgA nephropathy.

## Participants and methods

### Participants

All 11 probands referred consecutively with familial IgA nephropathy from teaching hospitals between 2014 and 2022 were studied. Each family included at least one member with biopsy-proven disease and another person considered affected because of hematuria (>trace) or proteinuria (≥1+) on dipstick of freshly collected urine specimens (Ames, Germany), impaired kidney function (eGFR < 90 mL/min/1.73 m^2^) without another obvious cause, or biopsy evidence of IgA nephropathy. End-stage Kidney failure (eGFR<15 mL/min/1.73 m^2^; dialysis-dependence or a current kidney transplant) was noted. The renal biopsies themselves were not available for study.

Furthermore, 39 consecutive unrelated patients with biopsy-proven IgA nephropathy but without another affected family member with IgA nephropathy or kidney disease (‘sporadic’ cases) were also examined. People with a secondary cause of IgA nephropathy (e.g., liver disease, etc.) were excluded.

A further five people with biopsy-proven IgA vasculitis and nine with sporadic glomerulonephritis [lupus nephritis in eight; Focal and Segmental glomerulosclerosis (FSGS) in one] were also studied.

This project was approved by the Austin Health Human Research Ethics Committee (HREC/16/Austin/538) in accordance with the principles of the Declaration of Helsinki, and all participants provided written informed consent for their involvement.

### Whole exome sequencing and variant filtering

Participants provided peripheral blood from which DNA was extracted using a QIAamp DNA Blood Mini Kit (Qiagen, Netherlands) and 50 ng of purified DNA underwent Whole Exome Sequencing (WES) using libraries prepared with the SureSelect XT Clinical Research Exome v2 target enrichment kits (Agilent Technologies, CA, USA) on an Illumina NovaSeq platform (California) at the Australian Genome Research Facility, Melbourne. Germline single-nucleotide variants (SNV) and short insertions/deletions (indels) were called using standard workflows based on the Genome Analysis Toolkit (GATK v4.4.0) (35). The variants were annotated using the Ensembl Variant Effect Predictor tool (36).

Variants were filtered for changes in 384 genes from the Genomics England panels for genetic kidney disease (Hematuria, Renal proteinuria, CAKUT Cystic kidney disease, and Renal ciliopathies, Kidney failure with no obvious cause) (Supplementary Table S1).^1^ HLA variants were not examined.

Genetic variants were filtered to identify rare variants [<30 alleles in gnomAD v2.1.1 (37)] that were pathogenic or likely pathogenic according to ACMG/AMP criteria (38) in Alamut^2^ based on the in silico prediction tools, Polyphen2, SIFT, and Mutation Taster (39–41), or pathogenic or likely pathogenic in ClinVar.^3^ The scores provided were calculated automatically by Alamut. Scores of at least 5, or those classified as likely pathogenic or pathogenic in ClinVar, were also noted. Two pathogenic variants in the same person were required for the diagnosis of a biallelic kidney disease.

## Results

### Familial IgA nephropathy (11 families)

**Figure 1:**
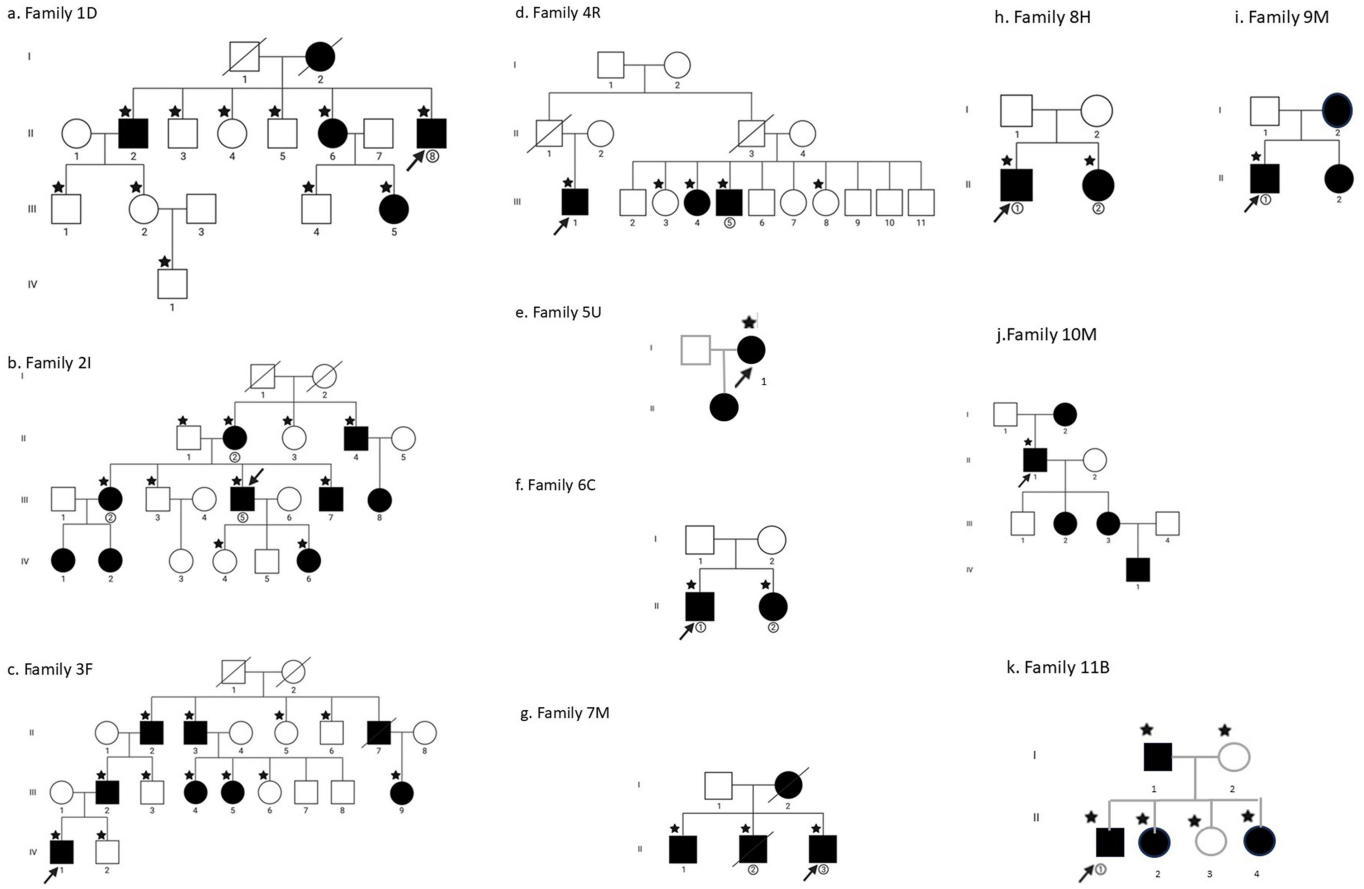
Family trees of the patients studied here. **(a–c)** Families 1–3; **(d–g)** Families 4–7; **(h–k)** Families 8–11. The index case is indicated by an arrow, and all family members are indicated by generation and birth order. Those with features of kidney disease (hematuria, proteinuria, and impaired kidney function) are demonstrated by the filled squares or circles. Those with a star underwent whole exome sequencing.

Eleven probands with familial IgA nephropathy and seven members of their families (median 4, range 2–9) were studied clinically and underwent WES (median 3, range 1–12) (Tables 1–3; Figures 1a–k). Nine families (82%) had at least one member with end-stage kidney failure.

**Table 1:** Evaluation of likely pathogenic variants in genes for genetic kidney disease in people with familial IgA nephropathy, sporadic IgA nephropathy, IgA vasculitis, or other autoimmune forms of glomerulonephritis.

Family	Gene	Disease and mode of inheritance	Sequence change	Alleles in gnomAD (v.2.1)	Alamut	ClinVar
1D proband	*C9*	AR predisposes to bacterial infections and autoimmune disease	NM_001737.5: c.162C > A; NP_001728.1: p.Cys54Ter (het)	247	Pathogenic (PVS1, PM2, PP5) score = 11	Pathogenic/ Likely pathogenic**; rs34000044
2I proband	*COL4A3*	AD, Alport syndrome	NM_000091.5: c.1184G > A; p.Gly395Glu (het)	None	Likely pathogenic (PM1, PM2, PP3, PP5) score = 6	Pathogenic/ Likely pathogenic**; rs1131691738
3F proband	*COL4A5*	XL Alport syndrome	NM_033380.3: c.4149_4150del; NP203699.1: p.138ArgfsTer39 (het)	None	Pathogenic (PVS1, PM2) score = 10	Not found
Other members of 3F	*COL4A4*	AD Alport syndrome	NM_000092.5: c.2906C > G; NP_000083.3: p.Ser969Ter (het)	17	Pathogenic (PVS1, PM2, PP5), Score = 11	Pathogenic **; rs35138315
7M proband	*HNF1B*	AD, HNF1B-nephropathy	NM_000458.4: c.1310C > T; NP_001291215.1: p.Pro437Leu (het)	None	VUS (PM2, PP5) score = 3	Likely pathogenic*
8H proband	*CHD7*	AD, Charge syndrome	NM_017780.4: c.3299G > A; P_060250.2: p.Arg1100His (het)	16	VUS (PM1, PM2, PP3) score = 5	Conflicting (Likely Benign or VUS)*; rs767259131
Other members of 11B	*INF2*	FSGS	NM_022489.4: c.*1 + 1G > C (het)	23	Pathogenic (PVS1, PM2) Score = 10	Conflicting* (VUS/Benign); Likely pathogenic^46^; rs758452999
Families where only the proband was tested						
5U proband	*COL4A4*	AD Alport syndrome	NM_000092.5: c.1396G > A; NP_000083.3: p.Gly466Arg (het)	5	VUS (PM2, PP3, PP5) score = 4	Pathogenic/ Likely pathogenic**; Rs:201859109
6C proband	*CLCN5*	XL, Dent disease	NM_001127899.4: c.1610G > A; NP_001121371.1: p.Arg537Gln (het)	None	VUS (PM1, PM2, PP3) Score = 5	Not found; not in HGMD
9M proband	*COL4A4*	AD Alport syndrome	NM_000092.5c. 1634G > T, p.Gly545Ala (het)	7,694	VUS (PM1, PM2, PP3) score = 5	VUS*; rs1800516
	*NPHS2*	FSGS (risk factor)	NM_014625.4 c.686G > A, p.Arg229Gln (het)	4,448	VUS (PM1, BP4) Score = 1	Conflicting (P, LP, VUS, B); rs.61747728
10M proband	*COL4A5*	XL Alport syndrome	NM_033380.3: c.1871G > A: NP_203699.1: p.Gly624Asp (het)	16	Likely pathogenic (PM1, PM2, PP3, PP5) score = 6	Pathogenic/ likely pathogenic**; Rs. 104,886,142
Individuals with sporadic IgA nephropathy (*n* = 39)						
S1	*COL4A3*	AD Alport syndrome	NM_000091.5: c.1855G > A P.Gly619Arg (het)	2	Pathogenic (PM1, PM2, PP3, PP5), score = 6	P/LP **; Rs.773515249
S47	*SLC7A9*	Cystinuria (AR and AD)	NM_014270.5 c.997C > T, p.Arg333Trp (het)	24	Likely pathogenic (PM1, PM2, PP3, PP5)	P/LP**; Rs.121908484
Individuals with IgA vasculitis (*n* = 5)						
No pathogenic variants detected in genes studied						
Individuals with other forms of glomerulonephritis (*n* = 9)						
G19	*SLC4A1*	Distal renal tubular acidosis type 1, AD	NM_000342.4 c.2102G > A, p.Gly701Asp (het)	10	Likely pathogenic (PM1, PM2, PP3, PP5) score = 6	Pathogenic**’ rs121912748

No disease-causing variant was found in family 4R.

* and ** refer to the ClinVar classification system; c.*1+1G>C refers to an upstream nucleotide variant G that has been substituted with C.

**Table 2:** Clinical features, family members tested, and likely pathogenic variants in genetic kidney disease genes in the proband and family members with familial IgA nephropathy.

Family	Proband (sex, age, ancestry)	Clinical features in the proband with biopsy-proven IgA glomerulonephritis	Total number of affected family members who were genetically tested	Number of first-degree family members sequenced	Pathogenic variant in the proband in the genetic kidney disease gene	Pathogenic variant in affected family members
1D	M, 46, N European	Hematuria, proteinuria, kidney failure, transplant	Four (brother also with biopsy-proven IgA disease, sister, niece)	Eleven	C9:p.Cys 24Ter	C9 variant in proband, three affected, and one unaffected family member
2I	M, 60, N European	Hematuria, proteinuria	Six (mother, uncle, cousin, sister, brother, daughter). Sister has a kidney transplant	Ten	p.Gly395Glu in *COL4A3* (AD Alport syndrome)	*COL4A3* variant segregated with hematuria in the proband and five affected family members
3F	M, 31, N European	Hematuria, impaired kidney function	Seven (father, grandfather, great uncle, three second cousins). Great uncle on dialysis	Twelve	p.Pro1384ArgfsTer39 in *COL4A5* only in proband (XL Alport syndrome)	*COL4A5* variant in proband only; *COL4A4* variant in great-uncle and two cousins. No pathogenic variant detected in the father or the grandfather of the proband
4R	M, 60, N European	Hematuria, proteinuria, impaired kidney function, and IgA vasculitis as a child	Three (proband and two cousins)	Five	None	None
5U	F, 64, S European	Hematuria, nearly normal kidney function	One (daughter also affected)	One	p.Gly466Arg in *COL4A4* LP (AD Alport syndrome)	Family members were not examined
6C	M, 51, N European	Kidney failure, transplant	One (sister affected mildly)	One	p.Arg537Gln in *CLCN5* (XL Dent disease)	Family members were not examined
7M	M, 38, S European	Hematuria, impaired kidney function	Proband and two brothers (both on dialysis)	Three	p.Pro437Leu in *HNF1B* (*HNF1B*-nephropathy)	*HNF1B* variant in all three affected brothers
8H	M, 56, N European	Hematuria, kidney failure, and a kidney transplant	Two (a sister also had biopsy-proven IgA glomerulonephritis)	Two	p.Arg1100His in *CHD7*, c.3299G > A (AD Hypo-gonadotrophic hypogonadism 5 with or without anosmia, CHARGE syndrome)	*CHD7* variant in the affected male only
9M	M, 55, N European	Hematuria, impaired kidney function	One (sister and mother also had hematuria)	One	p.Gly545Ala in *COL4A4* (AD Alport syndrome); p.Arg229Gln in *NPHP2* (FSGS)	Family members not examined
10M	M, 68, S European	Hematuria, kidney failure at 60, kidney transplant	One (mother, two daughters, and a grandson) was also affected	One	p.Gly624Asp in *COL4A5* (XL Alport syndrome)	Family members were not examined
11B	M, 35, N European	Hematuria, proteinuria, kidney failure, and kidney transplant	Four (father, two sisters)	Six	None	*INF2*: c.*1 + 1G > G (Pathogenic in HGMD) in the affected sister only

**Table 3:** Clinical features and pathogenic variants in affected members of families with IgA nephropathy.

Family and family member	Hematuria	Proteinuria	Impaired kidney function	Renal biopsy	Pathogenic variant
Family 1D					
II-2	YES			IgA gn	p.Cys54Ter in *C9*
II-6	YES	YES	YES		p.Cys54Ter in *C9*
II-8 (proband)	YES	YES	YES (eGFR<20 mL/min/1.73 m^2^), Transplant	IgA gn	p.Cys54Ter in *C9*
III-5	YES	NO	NO		p.Cys54Ter in *C9*
Family 2I					
II-2	YES		YES		p.Gly395Glu in *COL4A3*
II-4	YES	YES			p.Gly395Glu in *COL4A3*
III-2	YES	YES	YES, Transplant	IgA gn	p.Gly395Glu in *COL4A3*
III-5 (proband)	YES		NO	IgA gn	p.Gly395Glu in *COL4A3*
III-7	YES				p.Gly395Glu in *COL4A3*
IV-6	YES	YES	NO		p.Gly395Glu in *COL4A3*
Family 3F					
II-2	YES	YES			None detected
II-3	YES	YES	YES, Dialysis		p.Ser969Ter in *COL4A4*
III-2		YES		FSGS	None detected
III-4	YES	YES			p.Ser969Ter in *COL4A4*
III-5	YES				p.Ser969Ter in *COL4A4*
III-9	YES				None detected
IV-1 (proband)	YES	YES		IgA gn	p.Pro138ArgfsTer39 in *COL4A5*
Family 4R					
III-1 (proband)	YES	YES	YES	IgA vasculitis, Transplant	None detected
III-4	YES				None detected
III-5	YES				None detected
Family 5U					
I-1 (proband)	YES	NO	NO	IgA gn	p.Gly466Arg in *COL4A4*
Family 6C					
II-1 (proband)	YES	YES	YES, Transplant	IgA gn	p.Arg537Gln in *CLCN5*
II-2	YES	YES	NO		p.Arg537Gln in *CLCN5*
Family 7M					
II-1	YES	YES	YES, Dialysis	Diabetes, kidney cysts	p.Pro437Leu in *HNF1B*
II-2	YES	YES	YES, Dialysis	FSGS, diabetes, and kidney cysts	p.Pro437Leu in *HNF1B*
II-3 (proband)	YES	YES	YES	IgA gn, diabetes, kidney cysts	p.Pro437Leu in *HNF1B*
Family 8H					
II-1 (proband)	YES	YES	YES, Transplant	IgA gn	p.Arg1100His in *CHD7*
II-2	YES	NO	NO	IgA gn	None detected
Family 9M					
II-1 (proband)	YES	NO	NO	IgA gn	p.Gly545Ala in *COL4A4*, and p.Arg229Gln in *NPHP2* (both of which are risk factors)
II-2	YES				Not tested
					
Family 10M					
II-1 (proband)	YES	YES	YES, Transplant	IgA gn	p.Gly624Asp in *COL4A5*
III-2	YES				Not tested
III-3	YES				Not tested
IV-1	YES				Not tested
Family 11B					
I-1	YES	YES	YES		None detected
II-1 (proband)	YES	YES	YES, Transplant	IgA gn	None detected
II-2	YES	YES	YES	FSGS	c.*1 + 1G > C in *INF2*
II-4	YES				None detected

Of the 11 families studied, five (45%) had pathogenic variants previously associated with monogenic kidney diseases (2I, 3F, 5U, 7M, and 10M) (Tables 1, 2). Alport syndrome was found in the probands of four of these families (2I, 3F, 5U, and 10M), and the other had an *HNF1B* pathogenic variant and *HNF1B*-nephropathy.

Four further families (6C, 8H, 9M, and 11B) had interesting and possibly pathogenic variants, one additional family had two risk factors for kidney failure (9M), and one (1D) had a heterozygous variant in a gene (*C9*) previously associated with IgA nephropathy in the biallelic form.

Two of the families (3F and 11B) had members other than the proband who had a pathogenic (3F, p.Ser969Ter in *COL4A4*) or possible pathogenic variant (11B, c.1 + 1G > C in *INF2*) in genes affected in kidney disease (AD Alport syndrome, FSGS, respectively). Thus, this second variant in family 3F affected an Alport gene.

All disease-causing variants were heterozygous or hemizygous, rather than biallelic. Only two families (1D and 4R) had no suspected genetic kidney disease at all in the proband or a family member.

#### Families with genetic kidney disease

Five families had disease-causing variants. Four of the families had disease-causing variants in the Alport genes. Three families (2I, 5U, and 10M) each had one pathogenic variant in an Alport gene, and one (3F) had two pathogenic variants in Alport genes. The affected genes were *COL4A5* in two families (X-linked Alport syndrome), and *COL4A3* and *COL4A4* (AD Alport syndrome) in the others.

Family 3F had p.Pro1384ArgTer39 in *COL4A5* in the proband only, and three other 3F family members had a different heterozygous pathogenic variant (p.Ser969Ter in *COL4A4*) that was not present in the proband but segregated with hematuria. No family member had both variants. Proband 5U had a pathogenic variant in *COL4A4* (p.Gly466Arg) an Alamut score of 4, but which was considered Pathogenic in ClinVar. Furthermore, Proband 9M had the p.Gly545Ala variant in *COL4A4,* which may be associated with hematuria and kidney failure (42), but is generally not considered pathogenic. It was found together with an *NPHS2* variant (p.Arg229Gln) that results in FSGS in a biallelic disease and has been reported previously to worsen the effect of a monoallelic *COL4A3* or *COL4A4* variant (43, 44). Both the p.Gly545Ala in *COL4A4* and p.Arg229Gln in *NPHS2* are considered ‘risk factors’ for kidney failure but are not on their own associated with hematuria or proteinuria, respectively.

Furthermore, proband 7M had a heterozygous variant p.Pro437Leu in *HNF1B,* which, despite a pathogenicity score of 3 in Alamut, was considered likely pathogenic in ClinVar and consistent with the clinical phenotype of *HNF1B*-nephropathy in the patient and his two affected brothers, who all had diabetes, kidney cysts, infertility, and kidney failure.

#### Families with possibly disease-causing variants

Proband 1D had a heterozygous pathogenic variant in *C9,* which has been associated previously in the biallelic form with IgA nephropathy (26). This variant was also found in three other affected and one unaffected family member. While there is currently no known role for the monoallelic form of this variant in the pathogenesis of IgA nephropathy, at least one person has been reported with a low but detectable C9 level (45). This suggests a heterozygous variant that was not confirmed genetically.

Proband 6C had a possible variant in *CLCN5* (Dent disease), with a pathogenicity score of 5 in Alamut, and which was classified as a VUS. Inheritance for this gene is X-linked. The proband had developed kidney failure in his forties and his sister had hematuria and proteinuria, with mildly impaired kidney function but had not undergone WES. This variant was not present in ClinVar, LOVD, or HGMD. No more clinical details were available.

Proband 9M had variants in *COL4A4* and *NPHS2* (p.Gly545Ala, p.Arg229Gln, respectively) that are generally considered ‘risk factors’ for kidney failure rather than disease-causing, but the effect of the combination is unknown.

Proband 8H had a possibly pathogenic variant (p.Arg1100His) in the *CHD7* gene for Hypogonadotrophic hypogonadism 5 with or without anosmia/ CHARGE syndrome (Coloboma, Heart defects, Choanal atresia, Growth restriction, Genital abnormalities, and Ear abnormalities), which had a pathogenicity score of 5 in Alamut and was classified as a VUS. The patient had reflux nephropathy and low testosterone levels consistent with the variant being pathogenic, but none of the other facial, ocular, or cardiac features of CHARGE syndrome.

The proband in family 11B who had a kidney transplant had no pathogenic variant demonstrated but his sister with proteinuria had the c.*1 + 1G > C variant in *INF2* that was assessed as Pathogenic with a score of 10 in Alamut and as Conflicting in ClinVar (VUS and Benign). A review of genetic causes of the corresponding disease, Charcot–Marie–Tooth disease/FSGS, classified this variant as likely pathogenic (46). The sister’s clinical and renal biopsy features were consistent with FSGS.

### Sporadic IgA nephropathy (*n* = 39)

Thirty of the 39 individuals (77%) with sporadic IgA nephropathy had kidney failure, six (15%) had normal kidney function, and three (8%) had unknown kidney function.

Two (5%) had a heterozygous pathogenic variant consistent with genetic kidney disease (Table 3). These diseases were AD Alport syndrome (p.Gly619Arg in *COL4A3*); and AD Cystinuria (p.Arg333Trp in *SLC7A9*, Pathogenic/Likely pathogenic in ClinVar).

### IgA vasculitis (*n* = 5)

These included four individuals with childhood—and one with adult-onset disease, two of whom had kidney failure (40%). None had a pathogenic variant in a gene affected in genetic kidney disease (Table 3).

### Other autoimmune forms of glomerulonephritis (*n* = 9)

These included eight individuals with SLE and one with FSGS, where four (44%) had kidney failure, three (33%) had normal kidney function, and kidney function was unknown in two (22%). One person (11%) with normal kidney function had a heterozygous pathogenic *SLC4A1* variant consistent with the diagnosis of AD Distal renal tubular acidosis type 1, and there were no other genetic kidney diseases (Table 3).

## Discussion

This study demonstrated that families with IgA nephropathy commonly have an underlying genetic kidney disease that explains the familial occurrence. At least five, and probably more, of the 11 families described here had a pathogenic variant in a kidney disease-causing gene. Four families had pathogenic variants in the Alport genes, and five different Alport variants were detected. Genetic kidney disease was also found in 5% of individuals with sporadic IgA nephropathy, which was much less common than in the families.

Genetic kidney disease was found more often in this study than reported previously, in part because family members were tested and sometimes had a pathogenic variant not present in the index case. Our cohort was unbiased since the probands were those referred consecutively with familial IgA nephropathy over the period of review. Underlying genetic kidney disease in familial IgA nephropathy is likely to be even more common than demonstrated here because WES was used for genetic testing but detects only 80% of pathogenic changes (47–49) and sometimes overlooks large deletions, deep splicing changes and variants in alternative disease-causing genes.

These observations suggest that the mesangial IgA deposits, which are normally present in 20% of the population, coexist with genetic kidney disease (15, 16) and contribute to the more severe clinical course seen in familial IgA nephropathy (11, 12).

The genetic kidney diseases identified in familial IgA nephropathy reflect the commonest seen, namely, AD and XL Alport syndrome, and AD Tubulointerstitial kidney disease (ADTKD) (48). Another common disease, AD Polycystic Kidney Disease, was probably excluded based on family history and renal imaging. However, additional pathogenic variants in ADTKD-*HNF1B* and ADTKD-*MUC1* might have been missed because WES often does not recognize the large deletions that occur in *HNF1B* and the typical *MUC1* frameshift mutations that require targeted testing.

None of the genetic diagnoses demonstrated here were suspected before testing. In particular, the cysts in Family 7M with ADTKD-*HNF1B* nephropathy were only recognized on reverse phenotyping. Our cohort included two males with XL Alport syndrome, but neither had the typical hearing loss nor ocular abnormalities. The *COL4A5* p.Gly624Asp variant in Family 10M is associated with mild disease, late-onset kidney failure, and no extrarenal features. AD Alport syndrome was the commonest genetic diagnosis (32) but also was not associated with hearing loss or ocular abnormalities (50). Although the glomerular basement membrane is typically thinned in AD or lamellated in XL Alport syndrome, these features were not helpful diagnostically in this cohort. This was because electron microscopy of the kidney biopsy is not routinely performed in Australia; and in late stage kidney disease, the glomeruli are often too scarred to identify the membrane changes typical of Alport syndrome.

While each proband in the cohort had biopsy-proven IgA nephropathy, the diagnosis was often made clinically in other family members. In retrospect, the finding of hematuria, proteinuria, or impaired kidney function may have indicated a genetic kidney disease rather than IgA nephropathy. Indeed, this study found that the proband may have had IgA nephropathy but not the genetic disease present in other family members.

Familial IgA nephropathy is typically found in multiple members of a generation and in several generations, consistent with AD or XL inheritance. Our findings of coexisting genetic diseases with AD or XL inheritance were consistent with this. Autosomal recessive (AR) kidney diseases, such as FSGS, and the ciliopathies and tubulopathies, which are generally rarer, and more likely to occur sporadically, were not identified in this cohort. Any sex-based difference in severity may not be obvious with later onset disease and in families comprising mainly boys or affected females (51).

Genetic kidney disease occurred in 5% of our series of sporadic cases of IgA nephropathy, which was much less often than in familial disease. However, many sporadic cases also had kidney failure and a consequently increased risk of genetic disease (52). Coexisting genetic kidney disease was also present in a patient with autoimmune glomerulonephritis, but not in IgA vasculitis.

The prevalence of kidney failure in our cohorts of familial and sporadic IgA nephropathy may have increased the risk of genetic kidney disease, which occurs in up to 20% of those with kidney failure (52). Even AD Alport syndrome, where kidney function is typically normal, is associated with worse function in IgA nephropathy (11, 12).

IgA vasculitis was studied here because its mesangial IgA deposits resemble those found in IgA nephropathy, and its childhood onset suggested a genetic basis. However, no coexisting genetic kidney disease was found with IgA vasculitis, and an association with HLA type has been better substantiated (22).

The strengths of this study were the unbiased cohort with familial IgA nephropathy; the additional sequencing of members of most families; examination of a cohort with sporadic biopsy-proven IgA nephropathy; investigation of a large panel of genes associated with genetic kidney disease; and the use of rigorous filtering criteria and confirmation of pathogenicity in Alamut and ClinVar.

This study’s limitations were that many index cases had kidney failure, where genetic disease is more common anyway (52); clinical information, apart from hematuria, proteinuria and kidney failure, was limited and follow up was often incomplete; variants themselves were not confirmed independently with an alternate sequencing method; the original kidney biopsies were not available for further analysis, and in most cases the diagnosis of IgA nephropathy was made on a biopsy in one family member and presumed in others with kidney abnormalities. Further, pathogenic variants might have been overlooked because the regions of some genes were not adequately covered by WES.

In conclusion, many, and possibly most, familial IgA nephropathy results from IgA deposits and coexisting genetic kidney disease, especially the more common AD and XL Alport syndrome. Sometimes, genetic kidney disease is not detected in the proband but is present in another family member with IgA nephropathy. Genetic kidney disease also occurs in sporadic IgA nephropathy with kidney failure. Individuals with familial IgA nephropathy should be offered genetic testing, and family members should only be used as kidney donors if a genetic kidney disease has been excluded. Further, more sensitive testing may demonstrate that all cases of familial IgA nephropathy result from co-existing genetic kidney disease.

## Data Availability

The raw data supporting the conclusions of this article will be made available by the authors, without undue reservation.

## Footnotes

1
https://panelapp.genomicsengland.co.uk/

2
https://www.sophiagenetics.com/sophia-ddm-for-genomics/alamut-visual-plus/

3
https://www.ncbi.nlm.nih.gov/clinvar/

## Supplementary material

SUPPLEMENTARY TABLE S1 Candidate gene lists.

## References

[1] D'Amico G. The commonest glomerulonephritis in the world: IgA nephropathy. Q J Med. (1987) 64:709–27. 3329736

[2] Schena FP Nistor I. Epidemiology of IgA nephropathy: a global perspective. Semin Nephrol. (2018) 38:435–42. doi: 10.1016/j.semnephrol.2018.05.013, 30177015

[3] D'Amico G. Natural history of idiopathic IgA nephropathy: role of clinical and histological prognostic factors. Am J Kidney Dis. (2000) 36:227–37. doi: 10.1053/ajkd.2000.8966 10922300

[4] Zhang L Liu X Pascoe EM Badve SV Boudville NC Clayton PA . Long-term outcomes of end-stage kidney disease for patients with IgA nephropathy: a multi-centre registry study. Nephrology. (2016) 21:387–96. doi: 10.1111/nep.12629, 26393772

[5] Suzuki H Kiryluk K Novak J Moldoveanu Z Herr AB Renfrow MB . The pathophysiology of IgA nephropathy. J Am Soc Nephrol. (2011) 22:1795–803. doi: 10.1681/ASN.2011050464, 21949093 PMC3892742

[6] Kim CJ Woo YJ Kook H Choi YY Ma JS Hwang TJ. Henoch-Schonlein purpura nephritis associated with Epstein-Barr virus infection in twins. Pediatr Nephrol. (2004) 19:247–8. doi: 10.1007/s00467-003-1387-7, 14677057

[7] Hiki Y Odani H Takahashi M Yasuda Y Nishimoto A Iwase H . Mass spectrometry proves under-O-glycosylation of glomerular IgA1 in IgA nephropathy. Kidney Int. (2001) 59:1077–85. doi: 10.1046/j.1523-1755.2001.0590031077.x, 11231363

[8] Tomana M Novak J Julian BA Matousovic K Konecny K Mestecky J. Circulating immune complexes in IgA nephropathy consist of IgA1 with galactose-deficient hinge region and antiglycan antibodies. J Clin Invest. (1999) 104:73–81. doi: 10.1172/JCI5535, 10393701 PMC408399

[9] Matsuda M Shikata K Wada J Sugimoto H Shikata Y Kawasaki T . Deposition of mannan binding protein and mannan binding protein-mediated complement activation in the glomeruli of patients with IgA nephropathy. Nephron. (1998) 80:408–13. doi: 10.1159/000045212, 9832639

[10] Scolari F Amoroso A Savoldi S Mazzola G Prati E Valzorio B . Familial clustering of IgA nephropathy: further evidence in an Italian population. Am J Kidney Dis. (1999) 33:857–65. doi: 10.1016/s0272-6386(99)70417-8, 10213640

[11] Sato Y Tsukaguchi H Higasa K Kawata N Inui K Linh TNT . Positive renal familial history in IgA nephropathy is associated with worse renal outcomes: a single-center longitudinal study. BMC Nephrol. (2021) 22:230. doi: 10.1186/s12882-021-02425-8, 34147067 PMC8214250

[12] Shi M Yu S Ouyang Y Jin Y Chen Z Wei W . Increased lifetime risk of ESRD in familial IgA nephropathy. Kidney Int Rep. (2021) 6:91–100. doi: 10.1016/j.ekir.2020.10.015, 33426388 PMC7783566

[13] Li GS Zhang H Lv JC Shen Y Wang HY. Variants of *C1GALT1* gene are associated with the genetic susceptibility to IgA nephropathy. Kidney Int. (2007) 71:448–53. doi: 10.1038/sj.ki.5002088 17228361

[14] Kiryluk K Li Y Sanna-Cherchi S Rohanizadegan M Suzuki H Eitner F . Geographic differences in genetic susceptibility to IgA nephropathy: GWAS replication study and geospatial risk analysis. PLoS Genet. (2012) 8:e1002765. doi: 10.1371/journal.pgen.1002765, 22737082 PMC3380840

[15] Waldherr R Rambausek M Duncker WD Ritz E. Frequency of mesangial IgA deposits in a non-selected autopsy series. Nephrol Dial Transplant. (1989) 4:943–6. doi: 10.1093/ndt/4.11.943 2516884

[16] Suzuki K Honda K Tanabe K Toma H Nihei H Yamaguchi Y. Incidence of latent mesangial IgA deposition in renal allograft donors in Japan. Kidney Int. (2003) 63:2286–94. doi: 10.1046/j.1523-1755.63.6s.2.x, 12753320

[17] Varis J Rantala I Pasternack A Oksa H Jantti M Paunu ES . Immunoglobulin and complement deposition in glomeruli of 756 subjects who had committed suicide or met with a violent death. J Clin Pathol. (1993) 46:607–10. doi: 10.1136/jcp.46.7.607, 8157744 PMC501386

[18] Feehally J Farrall M Boland A Gale DP Gut I Heath S . HLA has strongest association with IgA nephropathy in genome-wide analysis. J Am Soc Nephrol. (2010) 21:1791–7. doi: 10.1681/ASN.2010010076, 20595679 PMC3013538

[19] Gharavi AG Kiryluk K Choi M Li Y Hou P Xie J . Genome-wide association study identifies susceptibility loci for IgA nephropathy. Nat Genet. (2011) 43:321–7. doi: 10.1038/ng.787, 21399633 PMC3412515

[20] Yu XQ Li M Zhang H Low HQ Wei X Wang JQ . A genome-wide association study in Han Chinese identifies multiple susceptibility loci for IgA nephropathy. Nat Genet. (2011) 44:178–82. doi: 10.1038/ng.1047, 22197929

[21] Kiryluk K Li Y Scolari F Sanna-Cherchi S Choi M Verbitsky M . Discovery of new risk loci for IgA nephropathy implicates genes involved in immunity against intestinal pathogens. Nat Genet. (2014) 46:1187–96. doi: 10.1038/ng.3118, 25305756 PMC4213311

[22] Lopez-Mejias R Castaneda S Genre F Remuzgo-Martinez S Carmona FD Llorca J . Genetics of immunoglobulin-A vasculitis (Henoch-Schönlein purpura): an updated review. Autoimmun Rev. (2018) 17:301–15. doi: 10.1016/j.autrev.2017.11.024, 29353097

[23] Ellinghaus D Jostins L Spain SL Cortes A Bethune J Han B . Analysis of five chronic inflammatory diseases identifies 27 new associations and highlights disease-specific patterns at shared loci. Nat Genet. (2016) 48:510–8. doi: 10.1038/ng.3528, 26974007 PMC4848113

[24] International Genetics of Ankylosing Spondylitis C Cortes A Hadler J Pointon JP Robinson PC Karaderi T . Identification of multiple risk variants for ankylosing spondylitis through high-density genotyping of immune-related loci. Nat Genet. (2013) 45:730–8. doi: 10.1038/ng.2667, 23749187 PMC3757343

[25] Milillo A La Carpia F Costanzi S D'Urbano V Martini M Lanuti P . A *SPRY2* mutation leading to MAPK/ERK pathway inhibition is associated with an autosomal dominant form of IgA nephropathy. Eur J Hum Genet. (2015) 23:1673–8. doi: 10.1038/ejhg.2015.52, 25782674 PMC4795196

[26] Yoshioka K Takemura T Akano N Okada M Yagi K Maki S . IgA nephropathy in patients with congenital C9 deficiency. Kidney Int. (1992) 42:1253–8. doi: 10.1038/ki.1992.412, 1453611

[27] Maillard N Wyatt RJ Julian BA Kiryluk K Gharavi A Fremeaux-Bacchi V . Current understanding of the role of complement in IgA nephropathy. J Am Soc Nephrol. (2015) 26:1503–12. doi: 10.1681/ASN.2014101000, 25694468 PMC4483595

[28] Li Y Groopman EE D'Agati V Prakash S Zhang J Mizerska-Wasiak M . Type IV collagen mutations in familial IgA nephropathy. Kidney Int Rep. (2020) 5:1075–8. doi: 10.1016/j.ekir.2020.04.011, 32647767 PMC7335950

[29] Stapleton CP Kennedy C Fennelly NK Murray SL Connaughton DM Dorman AM . An exome sequencing study of 10 families with IgA nephropathy. Nephron. (2020) 144:72–83. doi: 10.1159/000503564, 31865346

[30] Liu JW Wang P Huang J Nie XJ Zhao F Chen LZ . Genetic variants of familial hematuria associated genes in three families with hematuria with probands initially diagnosed as IgA nephropathy. Zhonghua Er Ke Za Zhi. (2019) 57:674–9. doi: 10.3760/cma.j.issn.0578-1310.2019.09.006, 31530352

[31] Paterson AD Liu XQ Wang K Magistroni R Song X Kappel J . Genome-wide linkage scan of a large family with IgA nephropathy localizes a novel susceptibility locus to chromosome 2q36. J Am Soc Nephrol. (2007) 18:2408–15. doi: 10.1681/ASN.2007020241, 17634434

[32] Gibson J Fieldhouse R Chan MMY Sadeghi-Alavijeh O Burnett L Izzi V . Prevalence estimates of predicted pathogenic *COL4A3-COL4A5* variants in a population sequencing database and their implications for Alport syndrome. J Am Soc Nephrol. (2021) 32:2273–90. doi: 10.1681/ASN.2020071065, 34400539 PMC8729840

[33] Nagamine K Peterson P Scott HS Kudoh J Minoshima S Heino M . Positional cloning of the *APECED* gene. Nat Genet. (1997) 17:393–8. doi: 10.1038/ng1297-393, 9398839

[34] Finnish-German *APECED* Consortium. An autoimmune disease, *APECED*, caused by mutations in a novel gene featuring two PHD-type zinc-finger domains. Nat Genet. (1997) 17:399–403. doi: 10.1038/ng1297-399 9398840

[35] DePristo MA Banks E Poplin R Garimella KV Maguire JR Hartl C . A framework for variation discovery and genotyping using next-generation DNA sequencing data. Nat Genet. (2011) 43:491–8. doi: 10.1038/ng.806, 21478889 PMC3083463

[36] McLaren W Gil L Hunt SE Riat HS Ritchie GR Thormann A . The Ensembl variant effect predictor. Genome Biol. (2016) 17:122. doi: 10.1186/s13059-016-0974-4, 27268795 PMC4893825

[37] Karczewski KJ Francioli LC Tiao G Cummings BB Alfoldi J Wang Q . The mutational constraint spectrum quantified from variation in 141,456 humans. Nature. (2020) 581:434–43. doi: 10.1038/s41586-020-2308-7, 32461654 PMC7334197

[38] Richards S Aziz N Bale S Bick D Das S Gastier-Foster J . Standards and guidelines for the interpretation of sequence variants: a joint consensus recommendation of the American College of Medical Genetics and Genomics and the Association for Molecular Pathology. Genet Med. (2015) 17:405–24. doi: 10.1038/gim.2015.30, 25741868 PMC4544753

[39] Adzhubei IA Schmidt S Peshkin L Ramensky VE Gerasimova A Bork P . A method and server for predicting damaging missense mutations. Nat Methods. (2010) 7:248–9. doi: 10.1038/nmeth0410-248, 20354512 PMC2855889

[40] Ng PC Henikoff S. SIFT: predicting amino acid changes that affect protein function. Nucleic Acids Res. (2003) 31:3812–4. doi: 10.1093/nar/gkg509, 12824425 PMC168916

[41] Schwarz JM Cooper DN Schuelke M Seelow D. MutationTaster2: mutation prediction for the deep-sequencing age. Nat Methods. (2014) 11:361–2. doi: 10.1038/nmeth.2890 24681721

[42] Ozdemir F Oygar DD Behlul A Atac S Bardak S Yukselis M Autosomal dominant tubulointerstitial kidney disease cosegregating with *COL4A4*:p.G545A in Turkish Cypriot families with kidney failure. doi: 10.21203/rs.3.rs2844330/v1

[43] Tonna S Wang YY Wilson D Rigby L Tabone T Cotton R . The R229Q mutation in *NPHS2* may predispose to proteinuria in thin-basement-membrane nephropathy. Pediatr Nephrol. (2008) 23:2201–7. doi: 10.1007/s00467-008-0934-7, 18726620

[44] Tsukaguchi H Sudhakar A Le TC Nguyen T Yao J Schwimmer JA . *NPHS2* mutations in late-onset focal segmental glomerulosclerosis: R229Q is a common disease-associated allele. J Clin Invest. (2002) 110:1659–66. doi: 10.1172/JCI16242 12464671 PMC151634

[45] Kanda E Shimamura H Tamura H Uchida S Terada Y Sakamoto H . IgA nephropathy with complement deficiency. Intern Med. (2001) 40:52–5. doi: 10.2169/internalmedicine.40.52, 11201372

[46] Vaeth S Christensen R Duno M Lildballe DL Thorsen K Vissing J . Genetic analysis of Charcot-Marie-tooth disease in Denmark and the implementation of a next generation sequencing platform. Eur J Med Genet. (2019) 62:1–8. doi: 10.1016/j.ejmg.2018.04.003 29653220

[47] Bullich G Domingo-Gallego A Vargas I Ruiz P Lorente-Grandoso L Furlano M . A kidney-disease gene panel allows a comprehensive genetic diagnosis of cystic and glomerular inherited kidney diseases. Kidney Int. (2018) 94:363–71. doi: 10.1016/j.kint.2018.02.027, 29801666

[48] Domingo-Gallego A Pybus M Bullich G Furlano M Ejarque-Vila L Lorente-Grandoso L . Clinical utility of genetic testing in early-onset kidney disease: seven genes are the main players. Nephrol Dial Transplant. (2022) 37:687–96. doi: 10.1093/ndt/gfab019, 33532864

[49] Hort Y Sullivan P Wedd L Fowles L Stevanovski I Deveson I . Atypical splicing variants in *PKD1* explain most undiagnosed typical familial ADPKD. NPJ Genom Med. (2023) 8:16. doi: 10.1038/s41525-023-00362-z, 37419908 PMC10328916

[50] Savige J. Heterozygous pathogenic *COL4A3* and *COL4A4* variants (autosomal dominant Alport syndrome) are common, and not typically associated with end-stage kidney failure, hearing loss, or ocular abnormalities. Kidney Int Rep. (2022) 7:1933–8. doi: 10.1016/j.ekir.2022.06.001, 36090501 PMC9458992

[51] Savige J Colville D Rheault M Gear S Lennon R Lagas S . Alport syndrome in women and girls. Clin J Am Soc Nephrol. (2016) 11:1713–20. doi: 10.2215/CJN.00580116, 27287265 PMC5012472

[52] Groopman EE Marasa M Cameron-Christie S Petrovski S Aggarwal VS Milo-Rasouly H . Diagnostic utility of exome sequencing for kidney disease. N Engl J Med. (2019) 380:142–51. doi: 10.1056/NEJMoa1806891, 30586318 PMC6510541

